# Radiological pleuroparenchymal fibroelastosis-like lesion in idiopathic interstitial pneumonias

**DOI:** 10.1186/s12931-021-01892-9

**Published:** 2021-11-11

**Authors:** Tomoyuki Fujisawa, Yasuoki Horiike, Ryoko Egashira, Hiromitsu Sumikawa, Tae Iwasawa, Shoichiro Matsushita, Hiroaki Sugiura, Kensuke Kataoka, Mikiko Hashisako, Hideki Yasui, Hironao Hozumi, Masato Karayama, Yuzo Suzuki, Kazuki Furuhashi, Noriyuki Enomoto, Yutaro Nakamura, Naoki Inui, Takafumi Suda

**Affiliations:** 1 Second Division, Department of Internal Medicine, Hamamatsu University School of Medicine, 1-20-1 Handayama Higashi-ku, Hamamatsu, 431-3192 Japan; 2 Department of Radiology, Faculty of Medicine, Saga University, Saga, Japan; 3 Department of Diagnostic Radiology, Sakai City Medical Center, Sakai, Japan; 4 Department of Radiology, Kanagawa Cardiovascular and Respiratory Center, Yokohama, Japan; 5 Department of Radiology, St.Marianna University School of Medicine, Kawasaki, Japan; 6 Department of Radiology, National Defense Medical College, Saitama, Japan; 7 Department of Respiratory Medicine and Allergy, Tosei General Hospital, Seto, Japan; 8 Department of Anatomic Pathology, Graduate School of Medical Sciences, Kyushu University, Fukuoka, Japan; 9 Department of Clinical Pharmacology and Therapeutics, Hamamatsu University School of Medicine, Hamamatsu, Japan

**Keywords:** Pleuroparenchymal fibroelastosis, Idiopathic interstitial pneumonias, Prognosis, IPF, Unclassifiable IIPs

## Abstract

**Background:**

Pleuroparenchymal fibroelastosis (PPFE) is characterised by predominant upper lobe pleural and subpleural lung parenchymal fibrosis. Radiological PPFE-like lesion has been associated with various types of interstitial lung diseases. However, the prevalence and clinical significance of radiological PPFE-like lesion in patients with idiopathic interstitial pneumonias (IIPs) are not fully understood. We aimed to determine the prevalence and clinical impact on survival of radiological PPFE-like lesion in patients with IIPs.

**Methods:**

A post-hoc analysis was conducted using data from the Japanese nationwide cloud-based database of patients with IIPs. All the patients in the database were diagnosed as having IIPs by multidisciplinary discussion. Patients diagnosed with idiopathic PPFE were excluded. Clinical data and chest computed tomography (CT) image of 419 patients with IIPs were analysed. The presence of radiological PPFE-like lesion was independently evaluated by two chest radiologists blind to the clinical data.

**Results:**

Of the 419 patients with IIPs, radiological PPFE-like lesions were detected in 101 (24.1%) patients, mainly in idiopathic pulmonary fibrosis (IPF) and unclassifiable IIPs, but less in idiopathic nonspecific interstitial pneumonia. Prognostic analyses revealed that radiological PPFE-like lesion was significantly associated with poor outcome in patients with IIPs, which was independent of age, IPF diagnosis and %FVC. In survival analyses, the patients with radiological PPFE-like lesions had poor survival compared with those without (log-rank, p < 0.0001). Subgroup analyses demonstrated that radiological PPFE-like lesion was significantly associated with poor survival both in patients with IPF and those with unclassifiable IIPs.

**Conclusion:**

Radiological PPFE-like lesion is a condition that could exist in IIPs, mainly in IPF and unclassifiable IIPs. Importantly, the radiological PPFE-like lesion is a non-invasive marker to predict poor outcome in patients with IIPs, which should be carefully considered in clinical practice.

**Supplementary Information:**

The online version contains supplementary material available at 10.1186/s12931-021-01892-9.

## Background

Idiopathic pleuroparenchymal fibroelastosis (iPPFE) is a condition of idiopathic interstitial pneumonias (IIPs) characterised by fibrosis of the pleura and subpleural lung parenchyma accompanied by elastosis of the alveolar walls, predominantly in the upper lobe [1, 2]. iPPFE was categorised as a rare IIPs in the current classification of the American Thoracic Society (ATS)/European Respiratory Society (ERS) Guidelines [3]. Its pathogenesis is not fully understood; however, recent studies have demonstrated that PPFE is observed in various conditions, including lung and bone marrow transplantation, connective tissue diseases (CTD) and history of anticancer/cytotoxic chemotherapy [1, 4–6]. Additionally, radiological PPFE-like lesion on high-resolution computed tomography (HRCT) has been reported in association with several forms of ILDs, including idiopathic pulmonary fibrosis (IPF), hypersensitivity pneumonitis and CTD-related interstitial lung disease (ILD) [7–11], with various prevalence and clinical implications.

The clinical significance of radiological PPFE-like lesions in patients with IIPs have not been fully understood. No large-scale study in multicenter cohort has assessed the prevalence and clinical impact of radiological PPFE-like lesions in patients with IIPs. Recently, we have developed the nationwide cloud-based integrated database with the clinical, radiological and pathological data of more than 500 patients with IIPs in Japan. Using the database, we have successfully performed web-based multidisciplinary discussion (MDD) for the 465 cases of IIPs and shown the utility of web-based MDD to increase the accuracy of IIP diagnosis [12]. The nationwide cloud-based database is the largest cohort of patients with MDD diagnosis of IIPs in Japan [12], in which the clinical, radiological and pathological data of the enrolled cases are available. Therefore, the database enables us to review radiological PPFE-like lesions on chest HRCT and to evaluate its clinical implications in large number of patients with IIPs.

The aims of the present study were to assess the prevalence of radiological PPFE-like lesion in patients with IIPs and to clarify its potential impact of survival using the nationwide large cohort of patients with IIPs.

## Methods

### Subject

A post-hoc analysis was conducted using the data from a retrospective cohort study of the Japanese nationwide cloud-based database of patients with IIPs for web-based MDD [12]. Previously, we built the nationwide cloud-based database containing clinical, radiological and pathological data of consecutive patients with institutional diagnosis of IIPs in 39 institutions (from April 2009 to March 2014) and web-based MDD system. Web-based MDD were performed for the 465 enrolled patients to make accurate diagnosis of IIPs [12]. The IIPs cohort in the nationwide cloud-based database represents a prevalence of IIPs in Japan. In this study, the 465 cases registered in the database were screened. The patients with MDD diagnosis of IIPs were eligible for inclusion in the absence of the following exclusion criteria: (1) insufficient data; (2) MDD diagnosis of iPPFE.

**Fig. 1:**
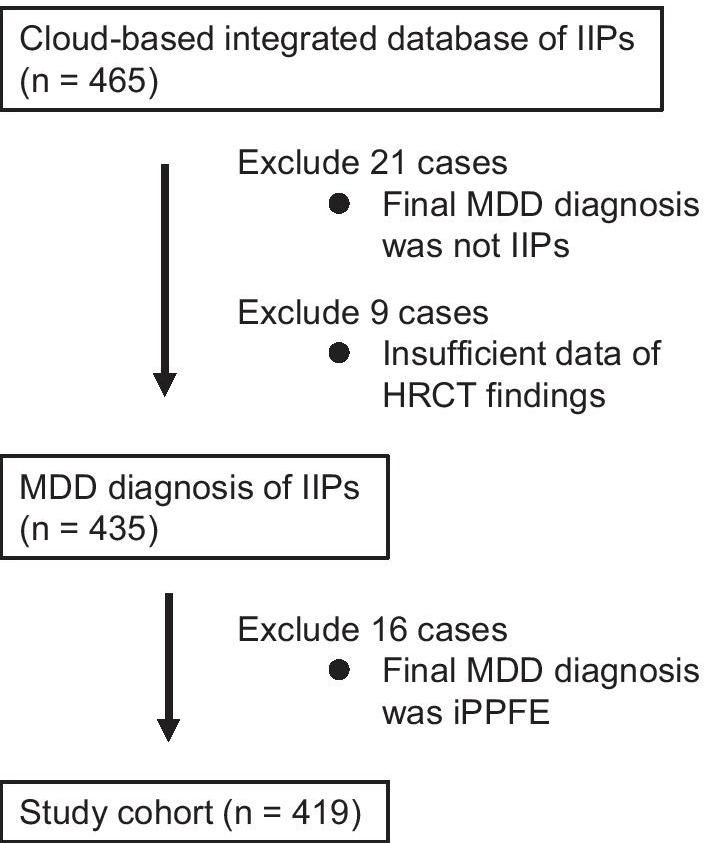
Flowchart of case inclusion in the study. Of the 465 patients with institutional diagnosis of IIPs in the nationwide cloud-based integrated database, 21 were diagnosed as having ILD other than IIPs by web-based MDD and were excluded. Because of insufficient data on HRCT findings, nine patients were excluded. Of the 435 patients with web-based MDD diagnosis of IIPs, 16 were diagnosed as having idiopathic PPFE and were excluded. Consequently, 419 patients with MDD diagnosis of IIPs except for iPPFE were enrolled in this study. *IIPs* idiopathic interstitial pneumonias; *ILD* interstitial lung disease; *MDD* multidisciplinary discussion; *HRCT* high-resolution computed tomography

The study flowchart is presented in Fig. 1. Of the 465 patients with institutional diagnosis of IIPs, 21 were diagnosed as having ILD other than IIPs by web-based MDD and were excluded from this study. Because of insufficient data on HRCT findings, nine patients were excluded. Sixteen patients diagnosed as having iPPFE by web-based MDD were also excluded. Consequently, 419 patients with MDD diagnosis of IIPs (except for iPPFE) were enrolled in this study. Diagnosis/classification of IIPs was categorised according to the ATS/ERS/Japanese Respiratory Society/Latin American Thoracic Association IPF statements [13] and ATS/ERS IIPs classification [3]. The categories included IPF, idiopathic nonspecific interstitial pneumonia (iNSIP), cryptogenic organizing pneumonia (COP), desquamative interstitial pneumonia (DIP)/respiratory bronchiolitis-ILD (RB-ILD), lymphoid interstitial pneumonia (LIP) and unclassifiable IIPs. This retrospective study was approved by the Institutional Review Board of the Hamamatsu University School of Medicine (approval number E14-360).

### Data collection

All the data were collected from the nationwide cloud-based database [12]. The patients’ clinical and HRCT data within 3 months before surgical lung biopsy (SLB), and whole slide images of biopsy specimens were retrospectively collected and registered in the database to performed web-based MDD [12]. The clinical data included age, sex, smoking history, serum Krebs von den Lungen-6 (KL-6), serum surfactant protein D (SP-D), serum lactate dehydrogenase, arterial oxygen pressure, arterial carbon dioxide tension (PaCO_2_), % FVC, %DLCO and survival outcomes.

### HRCT findings

**Fig. 2:**
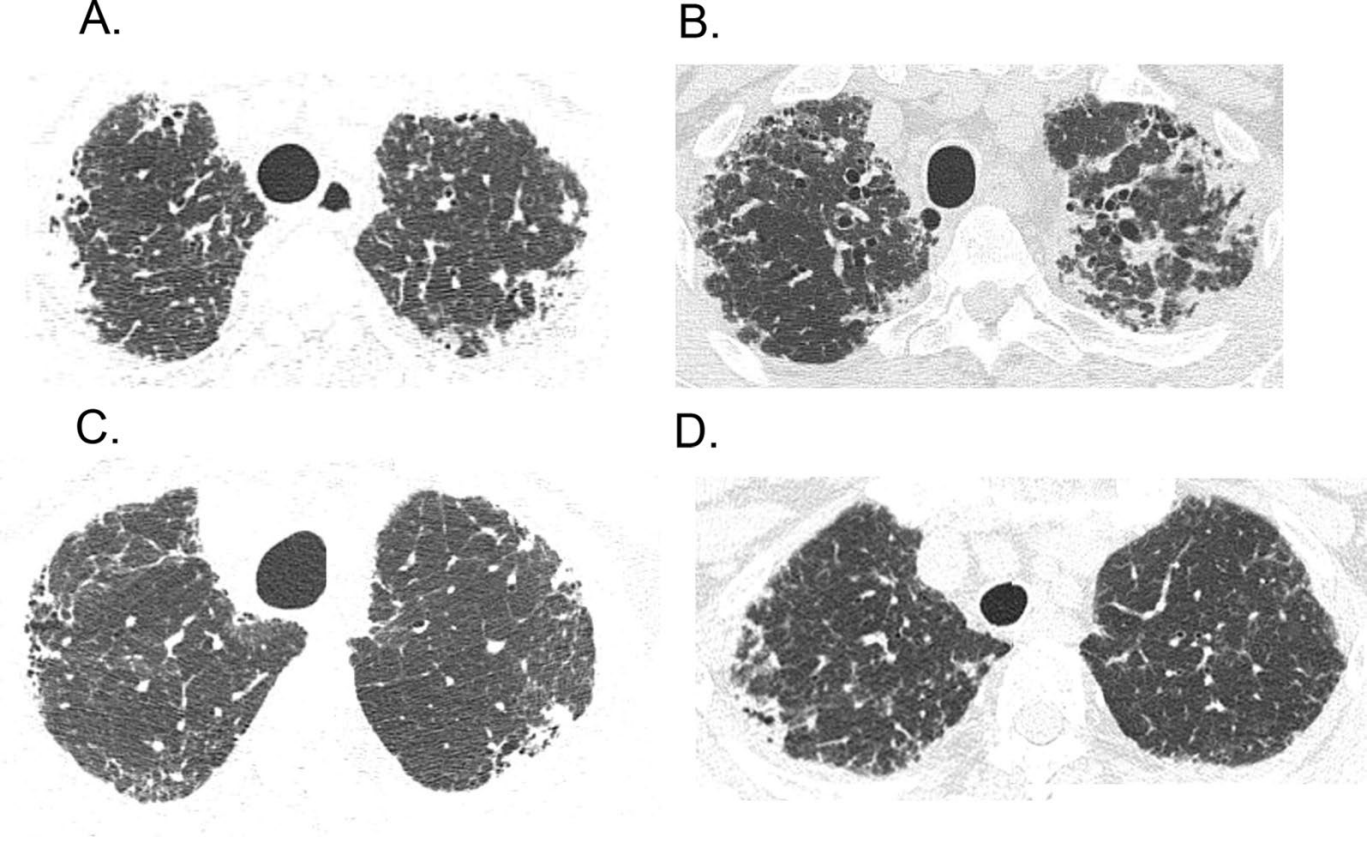
Example of chest HRCT images of radiological PPFE-like lesion. Axial HRCT images demonstrating bilateral, upper lobe, and subpleural dense consolidations with pleural thickening in IPF (**A**, **B**) and unclassifiable IIPs (**C**, **D**). *HRCT* high-resolution computed tomography; *PPFE*, pleuroparenchymal fibroelastosis; *IPF* idiopathic pulmonary fibrosis; *IIPs* idiopathic interstitial pneumonias

Chest HRCT images obtained within the 3 months prior to SLB were evaluated. The presence of radiological PPFE-like lesion of all the 419 cases were independently reviewed by two chest radiologists with 19 and 20 years of experience, respectively, who were blinded to the clinical data. The radiological PPFE-like lesion was defined on the basis of previous reports [1, 9, 14] as follows: bilateral, upper lobe and subpleural dense consolidation with or without pleural thickening observed below a line 1 cm from the apex of the lung, with a minimum width of 1 cm in contact with the pleura. Subpleural consolidations limited within 1 cm from apex of the lung were excluded from the definition of radiological PPFE-like lesion to discriminate pulmonary apical cap. The discordance of the presence of radiological PPFE-like lesion between the two radiologist was resolved by concordance of them. Representative images of PPFE-like lesion of HRCT are shown in Fig. 2.

Additionally, HRCT images had been evaluated by expert chest radiologists as described in the previous study [12]. HRCT images had been classified as usual interstitial pneumonia (UIP), possible UIP and inconsistent with UIP patterns according to the 2011 international guideline [13] for MDD diagnosis and assessed for the presence of the following findings: emphysema and honeycombing. The extent of emphysema was semi-quantitatively evaluated, and the area of emphysema of ≥ 10% of the whole lung area was considered present.

### Statistical analysis

Data were expressed as median (interquartile range) or number (%). Mann–Whitney U-test was used to compare continuous variables. Fisher’s exact test and the chi-square test were used to compare proportions among groups. Multiple testing was not used because of two-group comparison. Interobserver variability with respect to the presence of radiological PPFE-like lesion was evaluated using Cohen’s kappa (k) coefficient. Interobserver agreement was classified as follows: poor (k = 0–0.2), fair (k = 0.21–0.40), moderate (k = 0.41–0.60), good (k = 0.61–0.80), and excellent (k = 0.81–1.00). The observation period was calculated from the date of the first visit in each institution for an IIP to the last date of contact or the time of death. The vital status of the patients was ascertained on October 2017 for survival analysis. The Cox proportional hazard model was used for univariate and multivariate analyses to identify survival-associated variables. The variable of multivariate Cox-regression analyses in the whole cohort included diagnosis of IPF, pulmonary function (%FVC), both of which were considered as clinically important factors associated with mortality in IIPs [12, 15], as well as patients background (age, sex) and HRCT findings (PPFE-like lesion, emphysema and honeycombing). The Kaplan–Meier method was used to calculate the cumulative survival rate. The log-rank test was used to compare the survival rate between patient groups. Statistical analyses were performed using commercially available software (JMP version 9.0: SAS Institute, Inc., Cary, NC, USA). All tests were two-tailed, and a p value of < 0.05 was considered statistically significant.

## Results

### Patients’ characteristics and clinical data

This study included 419 patients with IIPs diagnosed by MDD. The enrolled patients’ characteristics and clinical data are summarised in Table 1. Radiological PPFE-like lesion was detected in 101 (24.1%) patients. Interobserver agreement for the presence of radiological PPFE-like regions was good (k = 0.67). IPF was the most prevalent MDD diagnosis in both groups, followed by unclassifiable IIPs. Patients with iNSIP had less radiological PPFE-like lesion, with radiological PPFE-like lesion detected in only three patients with iNSIP. Patients with radiological PPFE-like lesion were found to have higher levels of PaCO_2_ and SP-D than those without. On the HRCT findings, emphysema was less common in patients with radiological PPFE-like lesion than those without.

**Table 1:** Patients’ characteristics, MDD diagnosis and clinical data before surgical lung biopsy, and mortality

Variables	Total	Without PPFE	With PPFE	p value
	n = 419	n = 318	n = 101	
Age, years	65 (59, 70)	65 (58, 70)	67 (61, 71)	0.0319
Sex, male, n (%)	273 (65)	210 (66)	63(62)	0.5493
Never smokers, n (%)	147 (35)	102 (33)	45 (45)	0.0307
IPF, n (%)	199 (47)	145 (46)	54 (53)	
iNSIP, n (%)	44 (11)	41 (13)	3 (3)	
COP, n (%)	5 (1)	5 (1)	0 (0)	
DIP/RB-ILD, n (%)	9 (2)	9 (3)	0 (0)	
Unclassifiable IIPs, n (%)	162 (39)	118 (37)	44 (44)	
PaO_2_, mmHg	83.7 (76, 90)	83.5 (76, 90)	85.4 (77, 92)	0.2737
PaCO_2_, mmHg	40.9 (39, 44)	40.4 (38, 43)	42.7 (39, 45)	0.0006
LDH, U/mL	225 (199, 260)	226 (200, 264)	219 (191, 251)	0.0903
KL-6, U/mL	1088 (671, 1763)	1099 (677, 1894)	1040 (571, 1540)	0.1318
SP-D, ng/mL	198 (131, 322)	178 (118, 305)	248 (156, 373)	0.0013
%predicted FVC (%)	82.2 (70, 94)	83 (71, 96)	79 (67, 91)	0.0661
%predicted DLCO (%)	67.0 (53, 83)	66.2 (53, 81)	70.2 (54, 83)	0.2531
HRCT pattern				0.0041
UIP	38 (9)	25 (8)	13 (13)	
Possible UIP	226 (54)	162 (51)	64 (63)	
Inconsistent with UIP	155 (37)	131 (41)	24 (24)	
HRCT findings				
Emphysema	126 (30)	112 (35)	14 (14)	< 0.0001
Honeycombing	41 (10)	26 (8)	15 (15)	0.0632
Deceased	116 (28)	76 (24)	40 (40)	0.0027

Data are presented as n (%) or median (interquartile range)

*MDD* multidisciplinary discussion; *PPFE* pleuroparenchymal fibroelastosis; *IPF* idiopathic pulmonary fibrosis; *iNSIP* idiopathic nonspecific interstitial pneumonia; *COP* cryptogenic organizing pneumonia; *DIP* desquamative interstitial pneumonia; *RB-ILD* respiratory bronchiolitis-interstitial lung disease; *LIP* lymphoid interstitial pneumonia; *iPPFE* idiopathic pleuroparenchymal fibroelastosis; *IIPs* idiopathic interstitial pneumonias; *PaO*_*2*_ arterial oxygen tension; *PaCO*_*2*_ arterial carbon dioxide tension; *LDH* lactate dehydrogenase; *KL-6* Krebs von den Lungen-6; *SP-D* surfactant protein D; *FVC* forced vital capacity; *DLCO* diffusing capacity for carbon monoxide; *HRCT* high-resolution computed tomography; *UIP* usual interstitial pneumonia

Of 101 patients with radiological PPFE-like lesion, 8 patients (7.9%) developed lung cancer during their clinical courses, and 41 patients (12.9%) developed lung cancer in the 318 patients without radiological PPFE-like lesion. In terms of antifibrotic therapy, 41 patients (40.6%; 27 IPF, 13 unclassifiable IIPs, 1 iNSIP) with radiological PPFE-like lesion were treated with antifibrotic agents during their clinical courses. Of 318 patients without radiological PPFE-like lesion, 101 patients (31.8%; 70 IPF, 28 unclassifiable IIPs, 3 iNSIP) were treated with antifibrotic agents. In this study subject, only one patient with unclassifiable IIPs without radiological PPFE-like lesion underwent lung transplantation during the clinical course.

### Prognostic significance of radiological PPFE-like lesion in patients with IIPs

In the total cohort, 116 patients (28%) died, and mortality was significantly higher in patients with radiological PPFE-like lesion than those without (Table 1). To evaluate prognostic factors in patients with IIPs, Cox proportional hazard regression analyses were performed (Table 2). On the basis of univariate analyses, age, sex, IPF diagnosis, %FVC, UIP pattern on HRCT, PPFE-like lesion, emphysema and honeycombing were associated with poor prognosis. Multivariate analyses, however, demonstrated that age, IPF diagnosis, %FVC, the presence of PPFE-like lesion and emphysema on HRCT were significantly associated with poor outcome in patients with IIPs. When UIP pattern was included in multivariate analyses, we found that UIP pattern was not significantly associated with poor prognosis (Additional file 1: Table S1). As UIP pattern may be strongly related to IPF diagnosis and honeycombing, we performed multivariate analyses with UIP pattern and without IPF diagnosis and honeycombing. In this model, UIP pattern was significantly associated with poor prognosis (Additional file 1: Table S1). Radiological PPFE-like lesion was strongly associated with poor prognosis in every model, suggesting that presence of radiological PPFE-like lesion is an independent predictor of poor survival in patients with IIPs. Subgroup analyses of prognostic factors in patients with IPF or those with unclassifiable IIPs were performed. Both univariate and multivariate analyses revealed that radiological PPFE-like lesion was significantly associated with poor outcome in patients with IPF (Additional file 1: Table S2). Similarly, radiological PPFE-like lesion was independently related to poor prognosis in patients with unclassifiable IIPs (Additional file 1: Table S3).

**Table 2:** Analyses of prognostic factors in patients with IIPs (Cox proportional hazards model)

Variable	Univariate			Multivariate		
	HR	95% CI	p value	HR	95% CI	p value
Age (years)	1.05	1.03–1.08	< 0.0001	1.05	1.02–1.08	0.0001
Sex (male)	1.84	1.21–2.89	0.0036	1.32	0.82–2.20	0.2593
IPF diagnosis	2.47	1.70–3.65	< 0.0001	2.55	1.68–3.94	< 0.0001
PaO_2_ (mmHg)	1.00	0.99–1.01	0.5115			
PaCO_2_ (mmHg)	1.01	0.99–1.01	0.1163			
KL-6 (U/mL)	1.00	0.999–1.000	0.7180			
%FVC, %	0.99	0.977–0.996	0.0043	0.98	0.97–0.99	< 0.0001
%DLCO, %	0.99	0.979–0.998	0.0203			
UIP pattern	2.71	1.66–4.25	0.0002			
PPFE-like lesion	2.32	1.56–3.38	< 0.0001	2.54	1.64–3.89	< 0.0001
Emphysema	1.61	1.10–2.34	0.0141	1.99	1.29–3.09	0.0021
Honeycombing	2.99	1.86–4.63	< 0.0001	1.50	0.90–2.42	0.1177

*IPF* idiopathic pulmonary fibrosis; *PaO*_*2*_ arterial oxygen tension; *PaCO*_*2*_ arterial carbon dioxide tension; *KL-6* Krebs von den Lungen-6; *FVC* forced vital capacity; *DLCO* diffusing capacity for carbon monoxide; *UIP* usual interstitial pneumonia; *PPFE* pleuroparenchymal fibroelastosis; *HR* hazard ratio

### Survival analysis comparing patients with PPFE-like lesion and those without in IIPs

**Fig. 3:**
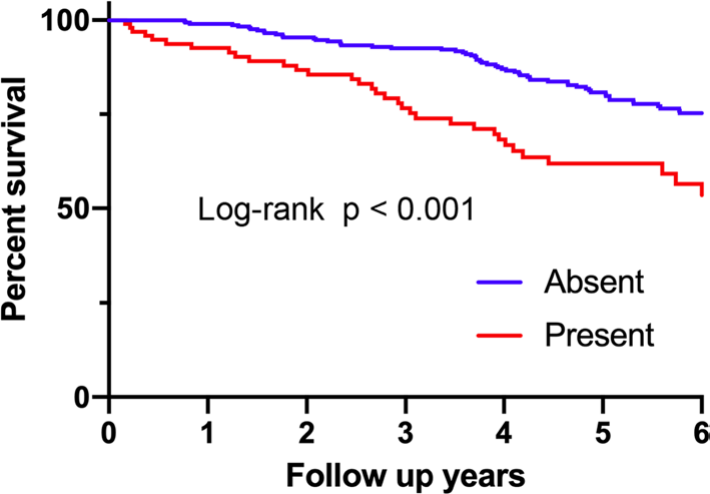
Survival comparison between patients with and without radiological PPFE-like lesion in the whole cohort. The survival of patients with radiological PPFE-like lesion was significantly worse than those without (log-rank, p < 0.0001). *PPFE* pleuroparenchymal fibroelastosis

**Fig. 4:**
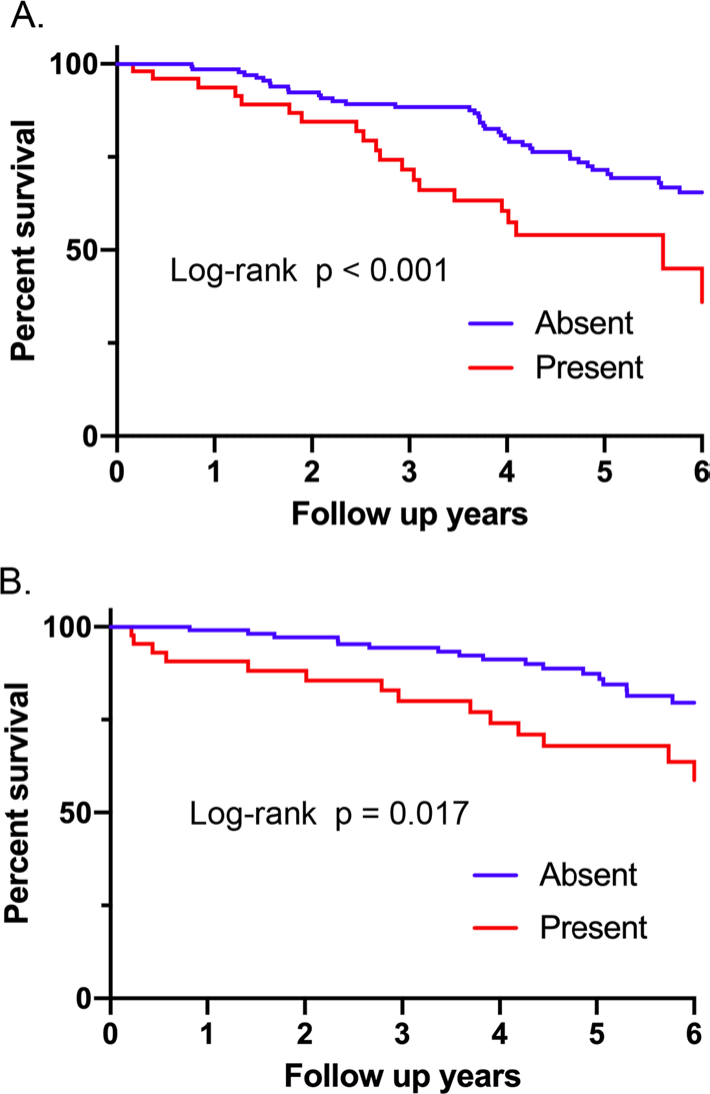
Subgroup survival comparison in IPF or unclassifiable IIPs between patientes with and without radiological PPFE-like lesions. **A** Patients with IPF with radiological PPFE-like lesion had a significantly worse survival than those without (log-rank, p = 0.0006). **B** Survival of the patients with unclassifiable IIPs with radiological PPFE-like lesion was significantly worse than those without (log-rank, p = 0.0166). *IPF* idiopathic pulmonary fibrosis; *IIPs* idiopathic interstitial pneumonias; *PPFE* pleuroparenchymal fibroelastosis

The survival curves comparing patients with radiological PPFE-like lesion and those without in the total cohort are shown in Fig. 3. The survival of patients with radiological PPFE-like lesion was significantly worse than those without. Median length of survival were 7.0 years for patients with radiological PPFE-like lesion and 9.9 years for those without. Subgroup analyses of survival curves in patients with MDD diagnosis of IPF or unclassifiable IIPs are shown in Fig. 4. Patients with IPF with radiological PPFE-like lesion were found to have a significantly poor survival than those without. Similarly, survival of patients with unclassifiable IIPs with radiological PPFE-like lesion was significantly worse than those without. Altogether, these results indicate that radiological PPFE-like lesion is an important prognostic factor for poor outcome in patients with IIPs.

## Discussion

This study demonstrated that radiological PPFE-like lesion was detected in disease entities of IIPs, mainly in patients with IPF and unclassifiable IIPs. Patients with radiological PPFE-like lesion had a worse survival compared with those without. Importantly, radiological PPFE-like lesion was an independent predictor of poor outcome in patients with IIPs.

This study is the first to evaluate the prevalence of radiological PPFE-like lesions in patients with IIP disease entities using the nationwide multicenter cohort of IIPs diagnosed by MDD. This study found that radiological PPFE-like lesion was detected in 24% of patients with IIPs. Former studies, using single-center cohort, have demonstrated the presence of radiological PPFE-like lesion in various types of ILD. Oda et al. showed that 10% of patients with biopsy-proven IPF met the radiological criteria of PPFE [7]. In a cohort of patients with CTD-related ILD, 19% were found to have radiological PPFE lesions [9]. Recently, Jacob et al. reported that radiological PPFE was identified in 23% of patients with hypersensitivity pneumonitis [8]. The findings of this study using the nationwide large cohort of IIPs indicate that radiological PPFE-like lesion is a condition that could exist in IIPs, mainly in IPF and unclassifiable IIPs.

Intriguingly, the prevalence of radiological PPFE-like lesion was different among IIPs disease entities. In this study, radiological PPFE-like lesions were detected in 27% of patients with IPF (54 of 199 patients) and unclassifiable IIPs (44 of 162 patients). Contrastingly, only three (6.8%) of the patients with iNSIP had radiological PPFE-like lesion. The presence of PPFE-like lesion in patients with IPF has been described in previous studies [7, 16]; however, no data were available about the prevalence of PPFE-like lesions in patients with iNSIP and those with unclassifiable IIPs. As all the patients enrolled in this study had been performed MDD for their diagnosis [12], IIP diagnoses of this study subjects were highly reliable. Thus, this study have revealed that radiological PPFE-like lesions are reasonably less common in patients with iNSIP. Contrary to iNSIP, radiological PPFE-like lesion was detected in a certain number of patients with unclassifiable IIPs. Unclassifiable IIPs are considered to include substantial heterogeneity in their clinical course and outcome and still have varied terminologies [17–19]. Further studies are needed to elucidate the prevalence and clinical implications of radiological PPFE-like lesion in unclassifiable IIPs.

Most importantly, this study found that the presence of radiological PPFE-like lesion is independently associated with poor prognosis in patients with IIPs. Furthermore, sub-group analyses of patients with IPF and those with unclassifiable IIPs revealed that patients with radiological PPFE-like lesion had a worse survival than those without, with statistical significance in both disease entities. In a cohort of 445 patients with IPF, Lee et al. reported that survival tended to be shorter in patients with radiological PPFE finding than those without PPFE [16]. Similarly, our previous study demonstrated that the presence of PPFE-like lesion on chest HRCT was an independent poor prognostic factor in patients with CTD-related ILD. Bonifazi et al. have recently shown that the prevalence of radiological PPFE feature was 18% in patients with systemic sclerosis, and the presence of PPFE feature was significantly associated with poor survival [10]. These findings including ours indicate that radiological PPFE-like lesion is a potential non-invasive marker to predict poor prognosis in various types of ILD.

The pathophysiology and the exact mechanism for forming radiological PPFE-like lesions ate still unknown. In this study, we did not pathologically examine radiological PPFE-like lesions in patients with IIPs, because lower lung fields were usually biopsied in the many cases for diagnosis of IIPs. In addition, since chest HRCT images after SLB was not collected in this cohort, it was difficult to validate whether the biopsy site was the same as the radiological PPFE-like lesion on chest HRCT images obtained before SLB. The assessment of pathological mechanisms of radiological PPFE-like lesions is an important topic, which should be addressed in future studies. Given that PPFE was histologically characterised by fibrosis of the pleura and subpleural lung parenchyma, radiological PPFE-like lesions may reflect severity of fibrosis in the lungs, which could be related to disease progression and prognoses of ILD. Due to clinical impact on survival of radiological PPFE-like lesion, clinicians should pay attention to the presence of the findings on HRCT as a non-invasive marker to predict worse prognosis in patients with ILD.

Distinction between radiological PPFE-like lesion and pulmonary apical cap is clinically important. PPFE-like lesion is usually progressive, whereas apical cap is asymptomatic and not progressive. Pulmonary apical cap is a type of fibroelastotic scar involving the lung apices, which represents an irregular density generally less than 5 mm located over the apex of lung [20]. In this study, radiological PPFE-like lesion was defined as bilateral, upper lobe and subpleural dense consolidations with or without pleural thickening observed below a line 1 cm from the apex of the lung to distinguish pulmonary apical cap. Using this definition, we found that radiological PPFE-like lesion was associated with a poor outcome in patients with IIPs. Recently, Sumikawa et al. evaluated significance of PPFE-like lesion in 207 patients with ILD including IIPs, hypersensitivity pneumonitis and CTD-related ILD [21]. In their study, the definition of PPFE-like lesion was different from ours. They defined PPFE-like lesion as subpleural consolidation associated with fibrosis in the upper lobe including apex of the lung, regardless of the extend of the caudal region of the apex of the lung. They reported that PPFE-like lesion was observed in more than 70% of the study subjects. In addition, the broad extent of PPFE like lesion under the aortic arch, which probably corresponds to our radiological PPFE-like lesion, were detected in 31% of the patients. Moreover, the broad extent of PPFE like lesion was significantly associated with poor survival; however, the small extent of PPFE-like lesion was not. In the present study, subpleural consolidations limited within 1 cm from apex of the lung were excluded from the definition of radiological PPFE-like lesion, which might be able to distinguish between PPFE-like lesion and pulmonary apical cap, and elucidate potential impact of radiological PPFE-like lesion on poor outcome in patients with IIPs.

This study has several limitations. First, a retrospective observational study was used. The observation period and treatment provided varied for each patient. The timing of drug administration (e.g., antifibrotic agents) depended on the patient’s clinical situations and was different in each case, which might affect the disease progression and prognosis. Second, all the patients enrolled in this study had undergone SLB, thus patients with typical clinical and radiological characteristics of IPF may be excluded, which may cause a selection bias. Third, sequential evaluation of radiological PPFE-like lesion on HRCT and clinical course (e.g., sequential FVC) was not available in the database. The precise distinction between radiological PPFE-like lesions and pulmonary apical cap might be difficult without serial clinical and radiological assessment to confirm disease progression. Further prospective multicenter observational studies are needed to validate the clinical implication of radiological PPFE-like lesion in patients with IIPs.

## Conclusions

In conclusion, this study demonstrated that radiological PPFE-like lesion was a condition that could exist in disease entities of IIPs, especially in IPF and unclassifiable IIPs. Radiological PPFE-like lesion was a significant marker to predict poor outcome in patients with IIPs. This study demonstrates the importance of evaluating radiological PPFE-like lesion for clinicians to provide optimal assessment of clinical outcome in patients with IIPs.

## Supplementary Information

**Additional file 1: Table S1.** Analyses of prognostic factors in patients with IIPs (Cox proportional hazards model). **Table S2.** Analyses of prognostic factors in patients with IPF (Cox proportional hazards model). **Table S3.** Analyses of prognostic factors in patients with unclassifiable IIPs (Cox proportional hazards model).

## Data Availability

The datasets used and/or analyzed during the current study are available from the corresponding author on reasonable request.

## Abbreviations

CT: Computed tomography
CTD: Connective tissue disease
COP: Cryptogenic organizing pneumonia
DIP: Desquamative interstitial pneumonia
HR: Hazard ratio
HRCT: High-resolution computed tomography
IIPs: Idiopathic interstitial pneumonias
ILD: Interstitial lung disease
iNSIP: Idiopathic nonspecific interstitial pneumonia
IPF: Idiopathic pulmonary fibrosis
iPPFE: Idiopathic pleuroparenchymal fibroelastosis
LIP: Lymphoid interstitial pneumonia
MDD: Multidisciplinary discussion
RB-ILD: Respiratory bronchiolitis-interstitial lung disease
SLB: Surgical lung biopsy
UIP: Usual interstitial pneumonia
