# Supplementary material for: Radiological pleuroparenchymal fibroelastosis-like lesion in idiopathic interstitial pneumonias
Source: Respir Res. 2021 Nov 11;22:290. doi: 10.1186/s12931-021-01892-9 (PMC8582158; doi:10.1186/s12931-021-01892-9)
Supplement: Supplementary file 1 — Additional file 1: Table S1. Analyses of prognostic factors in patients with IIPs (Cox proportional hazards model). Table S2. Analyses of prognostic factors in patients with IPF (Cox proportional hazards model). Table S3. Analyses of prognostic factors in patients with unclassifiable IIPs (Cox proportional hazards model). [file 12931_2021_1892_MOESM1_ESM.docx]

**Table S1. Analyses of prognostic factors in patients with IIPs (Cox proportional hazards model)**

|  | Multivariate | | | Multivariate | | |
| --- | --- | --- | --- | --- | --- | --- |
| Variable | HR | 95% CI | p value | HR | 95% CI | p value |
| Age (years) | 1.05 | 1.02–1.08 | 0.0001 | 1.05 | 1.02–1.78 | 0.0003 |
| Sex (male) | 1.31 | 0.81-2.18 | 0.2724 | 1.56 | 0.97-2.59 | 0.0756 |
| IPF diagnosis | 2.56 | 1.68–3.95 | <0.0001 |  |  |  |
| %FVC, % | 0.98 | 0.97–0.99 | <0.0001 | 0.98 | 0.97–0.99 | <0.0001 |
| UIP pattern | 0.43 | 0.09–4.01 | 0.4187 | 2.02 | 1.22–3.21 | 0.0043 |
| PPFE-like lesion | 2.57 | 1.66–3.94 | <0.0001 | 2.17 | 1.42–3.28 | 0.0025 |
| Emphysema | 2.01 | 1.30–3.12 | 0.0019 | 1.95 | 1.26–3.02 | 0.0003 |
| Honeycombing | 3.42 | 0.36–215.8 | 0.2563 |  |  |  |

**Table S2. Analyses of prognostic factors in patients with IPF (Cox proportional hazards model)**

|  | Univariate | | | Multivariate | | |
| --- | --- | --- | --- | --- | --- | --- |
| Variable | HR | 95% CI | p value | HR | 95% CI | p value |
| Age（years） | 1.06 | 1.03-1.10 | 0.0004 | 1.08 | 1.04-1.12 | < 0.0001 |
| Sex (male) | 1.25 | 0.73-2.30 | 0.4238 | 1.35 | 0.71-2.68 | 0.3584 |
| %FVC, % | 0.98 | 0.97-0.99 | 0.0007 | 0.97 | 0.96-0.98 | < 0.0001 |
| PPFE-like lesion | 2.38 | 1.41-3.90 | 0.0016 | 2.69 | 1.48-4.84 | 0.0015 |
| Emphysema | 1.06 | 0.65-1.69 | 0.8036 | 1.61 | 0.93-2.78 | 0.0891 |
| Honey combing | 2.28 | 1.35-3.73 | 0.0025 | 1.41 | 0.81-2.38 | 0.2221 |

IPF, idiopathic pulmonary fibrosis; FVC, forced vital capacity; PPFE, pleuroparenchymal fibroelastosis.

**Table S3. Analyses of prognostic factors in patients with unclassifiable IIPs (Cox proportional hazards model)**

|  | Univariate | | | Multivariate | | |
| --- | --- | --- | --- | --- | --- | --- |
| Variable | HR | 95% CI | p value | HR | 95% CI | p value |
| Age（years） | 1.03 | 0.99-1.07 | 0.1710 | 1.01 | 0.98-1.06 | 0.4630 |
| Sex (male) | 1.51 | 0.78-3.11 | 0.2275 | 1.11 | 0.49-2.60 | 0.8043 |
| %FVC, % | 0.99 | 0.97-1.01 | 0.2677 | 0.99 | 0.97-1.00 | 0.1513 |
| PPFE-like lesion | 2.17 | 1.12- 4.13 | 0.0224 | 2.26 | 1.08- 4.64 | 0.0304 |
| Emphysema | 2.58 | 1.32-4.99 | 0.0059 | 3.36 | 1.53-7.60 | 0.0026 |
| Honey combing | 1.57 | 0.25-5.18 | 0.5648 | 1.54 | 0.24-5.35 | 0.5833 |

IIPs, idiopathic interstitial pneumonias; FVC, forced vital capacity; PPFE, pleuroparenchymal fibroelastosis.
